# Moxifloxacin and Sulfamethoxazole-Based Nanocarriers Exhibit Potent Antibacterial Activities

**DOI:** 10.3390/antibiotics10080964

**Published:** 2021-08-11

**Authors:** Noor Akbar, Jasra Gul, Ruqaiyyah Siddiqui, Muhammad Raza Shah, Naveed Ahmed Khan

**Affiliations:** 1 College of Arts and Sciences, American University of Sharjah, University City, Sharjah 26666, United Arab Emirates; nakbar@aus.edu (N.A.); rsiddiqui@aus.edu (R.S.); 2 International Centre for Chemical and Biological Sciences, H.E.J. Research Institute of Chemistry, University of Karachi, Karachi 75270, Pakistan; jasra.gul123@yahoo.com (J.G.); raza.shah@iccs.edu (M.R.S.); 3 Department of Clinical Sciences, College of Medicine, University of Sharjah, University City, Sharjah 27272, United Arab Emirates

**Keywords:** antibiotic resistance, nanocarriers, Moxifloxacin, Sulfamethoxazole, cytotoxicity, niosomes

## Abstract

Antibiotic resistance is a major concern given the rapid emergence of multiple-drug-resistant bacteria compared to the discovery of novel antibacterials. An alternative strategy is enhancing the existing available drugs. Nanomedicine has emerged as an exciting area of research, showing promise in the enhanced development of existing antimicrobials. Herein, we synthesized nanocarriers and loaded these with available clinically approved drugs, namely Moxifloxacin and Sulfamethoxazole. Bactericidal activity against Gram-negative (*Serratia marcescens*, *Pseudomonas aeruginosa*, *Klebsiella pneumoniae*, and *Salmonella enterica*) and Gram-positive (methicillin-resistant *Staphylococcus aureus*, *Streptococcus pneumoniae*, and *Bacillus cereus*) bacteria was investigated. To characterize the nanocarriers and their drug-loaded forms, Fourier-transform infrared spectroscopy, dynamic light scattering, and atomic force microscopy were utilized. Antibacterial assays and hemolysis assays were carried out. Moreover, lactate dehydrogenase assays were performed to determine cytotoxicity against human cells. The results depicted the successful formation of drug–nanocarrier complexes. The potent antibacterial activities of the drug-loaded nanocarriers were observed and were significantly enhanced in comparison to the drugs alone. Hemolysis and cytotoxicity assays revealed minimal or negligible cytotoxic effects against human red blood cells and human cells. Overall, metronidazole-based nanocarriers loaded with Moxifloxacin and Sulfamethoxazole showed enhanced bactericidal effects against multiple-drug-resistant bacteria compared with drugs alone, without affecting human cells. Our findings show that drug-loaded nanocarriers hold promise as potent chemotherapeutic drugs against multiple-drug-resistant bacteria.

## 1. Introduction

The dramatic rise in antibiotic resistance demands the discovery of novel antibacterial agents [1]. In part, this is due to the misuse of antibiotics, which has resulted in the rise of multiple-drug-resistant (MDR) bacteria, which is a growing concern [2,3]. Resistant bacteria such as *Staphylococcus aureus*, *E. coli*, and *Pseudomonas aeruginosa* are some examples. Consequently, there is an urgent need to discover innovative antimicrobial compounds for the treatment of human and animal diseases due to resistant bacteria. In the past few decades, the World Health Organization (WHO) and the Centers for Disease Control and Prevention (CDC) have focused on finding solutions to combat antibiotic resistance [4]. Despite tremendous efforts, the emergence of MDR bacteria is being observed at an alarming pace. Currently, methicillin-resistant *Staphylococcus aureus* (MRSA), vancomycin-resistant *Enterococcus* (VRE), *Pseudomonas aeruginosa*, *Acinetobacter baumannii*, *Escherichia coli*, and *Klebsiella* *pneumonia* present a significant danger to public health, with social repercussions as well as a financial burden. Furthermore, bacteria that instigate food-borne illnesses, acute respiratory infections, and central nervous system and cutaneous infections also remain a major concern for human health. Thus, there is a clear and urgent need to discover novel antibacterials. Nonetheless, an alternative approach is to modify existing drugs to enhance their efficacy [5]. For the latter, several studies have depicted the potential application of nanotechnology against MDR bacteria [6,7,8,9,10]. Nanomaterials have distinctive properties that enable them to be used in biological and material sciences [11]. Nanomaterials (nanoparticles and nanocarriers) are considered safe and can penetrate cells/tissues safely due to their miniature size and increased surface area [12]. Nanocarriers have been used as vehicles for drug delivery. Studies have shown that nanocarriers can increase the bioavailability and therapeutic competence of drugs, while offering better accumulation at the target site [13,14]. The loading of drugs within the carrier materials (nanomaterials) can enhance their biological applications, including their antimicrobial activity [7,15].

Moxifloxacin (Mox), being a fluoroquinolone, has revealed an extended spectrum of antibacterial efficacy against pathogenic bacteria [16]. It has remarkable antibacterial efficacy against Gram-positive pathogenic cocci as well as retaining efficacy against Gram-negative bacteria [17]. Similarly, Sulfamethoxazole (Sulp) is a broad-spectrum antibacterial agent that is used currently to treat several bacterial infections [18]. The drug exhibits bacteriostatic effects by producing tetrahydrofolic acid, which is the active form of folic acid. It also arrests the synthesis of purines, thymidine, and bacterial DNA [18].

To determine whether the loading of the abovementioned drugs within nanomaterials can augment antibacterial efficacy, we loaded these drugs within nanocarriers and then tested them for their antibacterial activities using bactericidal experiments. Prior to determining their bioactivity, Fourier-transform infrared (FTIR), atomic force microscopy (AFM), zeta sizer, and zeta potential analysis were carried out to characterize these formulations. Hemolysis and lactate dehydrogenase (LDH) assays were performed to test the cytotoxicity of the drug-loaded nanocarriers against human cells and red blood cells. The loading of existing drugs with nanocarriers enhanced the potency of antibacterial efficacy, with marginal cytotoxic effects against human cells. These outcomes are significant and should encourage the utilization of novel antibacterial compositions in the clinical setting.

## 2. Materials and Methods

### 2.1. Synthesis of 2-(2-Methyl-5-nitro-1H-imidazol-1-yl)ethyldecanoate (DC-Met-10)

**Scheme 1:**
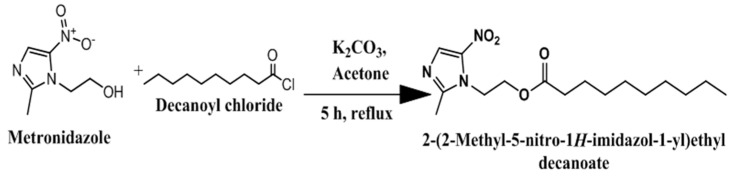
Scheme of DC-Met-10 synthesis. Preparation of drug-loaded DC-Met-10 niosomal formulation.

Synthesis of DC-Met-10 as outline in below was carried out by using a vigorously stirred solution of Metronidazole (500 mg; 2.921 mmol) in 25 mL acetone, refluxed at 60 °C for 30 min, followed by adding decanoyl chloride (0.62 mL; 3 mmol). Next, it was refluxed further for 10 h with constant stirring (Scheme 1). The progression of the reaction was monitored by thin-layer chromatography (TLC). After complete consumption of the starting material, the solvent was evaporated from the reaction mixture after cooling to room temperature. After removing the excess solvent, the reaction mixture was poured into chilled distilled water. The aqueous part was extracted with n-hexane (3 × 25 mL), the organic portions were combined, excess hexane was removed through rotary evaporation, and the product was purified by silica gel chromatography with an 8:2 (n-hexane: ethyl acetate) solvent system to obtain gummy liquid with a yield of 76% (Scheme 1). ^1^H-NMR (MeOD) ppm: 0.90 (t, 3H, CH_3_, *j* = 4 Hz), 1.26–1.28 (m, 12H, CH_2_), 1.46–1.54 (m, 2H, CH_2_), 2.2 (t, 2H, CH_2_-CO, *j* = 7.2 Hz), 2.50 (s, 3H, Heterocyclic CH_3_,), 4.42 (t, 2H, CH_2_-N, *j* = 5.2 Hz), 4.66 (t, 2H, CH_2_-O, *j* = 5.2 Hz), 7.92 (s, 1H, heterocyclic proton). Using EI-MS, the resultant ion peak appeared at 326.2 *m*/*z*.

Moxifloxacin and Sulfamethoxazole-loaded niosomal vesicle (Mox-Met-Lip and Sul-Met-Lip) from the synthesized amphiphile (DC-Met-10) was prepared as previously described [19]. Moxifloxacin-loaded niosomal vesicles were prepared by adding DC-Met-10 (100 mg), Moxifloxacin (100 mg), and cholesterol (50 mg) in a round-bottomed flask with 30 mL of methanol. Similarly, Sulfamethoxazole-loaded niosomal vesicles were prepared by adding DC-Met-10 (100 mg), Sulfamethoxazole (100 mg), and cholesterol (50 mg) in another round-bottomed flask with 30 mL of methanol, and solvent was evaporated using a rotary evaporator, leading to a thin film of niosomal vesicles. Using 100 mL of distilled water, these films were rehydrated in a bath sonicator at 30 °C for approx. 15 min, followed by sonication for 2 min at 25 °C with 5 sec on/off cycle to reduce the particle size, and suspensions were kept at 4 °C.

### 2.2. Zeta Potential, Size, Polydispersity Index, and Surface Morphology

Samples were characterized for surface morphology, zeta potential, size, and polydispersity index as described previously [7,20]. Using atomic force microscopy (AFM; Agilent 5500; Agilent Technologies, Santa Clara, CA, USA), the shapes of Met-Lip, Mox-Met-Lip, and Sul-Met-Lip were investigated as described earlier [7]. Moreover, techniques such as dynamic light scattering (DLS) using a zeta sizer analyzer were used to analyze the hydrodynamic diameter and PDI of these niosomal vesicles (Mox-Met-Lip, Sul-Met-Lip, and Met-Lip). These formulations were diluted with distilled water prior to determination of size. Zeta sizer dip cells were used to determine zeta potential.

### 2.3. Efficiency of Drug Entrapment 

Efficiency of drug entrapment was determined as previously described [19] using a UV–visible spectrophotometer (UV-240, Hitachi U-3200, Tokyo, Japan). Mox-Met-Lip and Sul-Met-Lip formulations of Moxifloxacin and Sulfamethoxazole were spun at 12,000 rpm for 30 min. Pellets containing drug were re-suspended in distilled water and subjected to UV spectrophotometry. Moxifloxacin and Sulfamethoxazole were detected at 293 nm and 267 nm. The efficiency of drug entrapment was determined as follows: EE% = (Amount of drug entrapped/Total amount of drug added) × 100.

### 2.4. FTIR Spectroscopy

To analyze the possible interaction of Moxifloxacin (Mox) with formulation excipients, pure Mox, drug excipients (Cholestrol), synthesized amphiphilic molecule (DC-Met-10), and drug-loaded niosomal formulations (Mox-Met-Lip, SUL-Met-Lip, and empty vesicle (Met-Lip)) were mixed with a KBr disc and pressed to form a self-supporting disk. Spectra were scanned in the range of 400–4000 cm^−1^ with an IR-470 spectrometer (Shimadzu, Kyoto, Japan) [7].

### 2.5. Hemolysis Assay

To determine the toxic effect of the synthesized amphiphilic compound (DC-Met-10) towards red blood cells, a hemolysis test was performed [21]. Briefly, cells were diluted with sodium citrate as an anti-coagulating agent in a 9:1 ratio and diluted with PBS (2.5 mL) in 1:1.25. Next, doses of DC-Met-10 were added, and samples were kept in water bath at 37 °C for approx. 30 min, followed by centrifugation at 900 rpm for 5 min. Finally, the supernatant was collected and the hemolysis ratio was calculated using a UV–vis spectrophotometer at 545 nm as follows:

(1) $$\mathrm{HR}\;\left(\%\right)=\frac{\left(\mathrm{As}-\mathrm{An}\right)}{\left(\mathrm{Ap}-\mathrm{An}\right)}\times 100$$

where (As) is the sample absorbance readout whereas (Ap) and (An) are the positive and negative control absorbance readout, respectively. (Positive control containing 0.2 mL of blood mixed with 10 mL of distilled water. Similarly, negative control containing 0.2 mL of blood mixed with 10 mL of PBS.)

### 2.6. Bacterial Cultures Used in this Study

Several MDR bacteria were used, including Gram-positive (methicillin-resistant *S. aureus* (MRSA), *B. cereus*, and *S. pneumoniae*) and Gram-negative (*P. aeruginosa*, *S. marcescens*, *S. enterica*, and *K. pneumoniae*) (Table 1). All bacterial isolates were from clinical samples. These bacteria were grown aerobically in nutrient broth (NB) at 37 °C overnight preceding experiments, as previously depicted [22,23].

### 2.7. Antibacterial Assays

Bactericidal assays were performed to determine the efficacy of drugs and drug-loaded nano-formulations against MDR bacteria [22,23]. Briefly, drugs alone, nanocarriers alone, and drug-loaded nanocarriers were kept with 1 × 10^6^ bacteria for 2 h at 37 °C. Next, ten-fold serial dilutions were accomplished with plating on nutrient agar plates. Plates were kept overnight at 37 °C. Finally, colonies were counted to estimate viable bacterial colony-forming units (c.f.u). For negative controls, bacteria were cultured alone in phosphate-buffered saline (PBS), while for the positive control, bacteria were treated with gentamicin (100 µg/mL). 

### 2.8. In Vitro Host Cell Cytotoxicity 

To elucidate the cytotoxic effects of drugs, nanocarriers, and drug-loaded nanocarriers, lactate dehydrogenase assays were performed as described earlier [22]. HeLa (Henrietta lacks cervical adenocarcinoma) monolayers were cultivated in 96-well plates up to 80–90% confluency. After this, monolayers were challenged with drugs alone, nanocarriers alone, and drug-loaded nanocarriers, and plates were placed at 37 °C at 5% CO_2_ and 95% humidity for 24 h. The following day, Triton X-100 (0.1%) was incorporated into positive control wells and plates were placed at 37 °C for 1 h. Subsequently, an equal amount of LDH^Plus^ kit reagent (Cytotoxicity Detection kit; Roche Diagnostics, Indianapolis, IN, USA) was mixed with equal volume of cell supernatant containing the enzyme (Lactate Dehydrogenase). HeLa cells in RPMI alone were utilized as negative control. The amount of liberated enzyme from HeLa cells was estimated by the given formula: Percent cytotoxicity = (value of sample − value of negative control value)/(value of positive control − value of negative control) × 100.

### 2.9. Statistical Asessment

For statistical assessment, the Graph Pad Prism software, version 8.0.2 (GraphPad, San Diego, CA, USA), was utilized. T-test (two-tailed distribution) *p* values were elucidated and *p* value ≤ 0.05 was denoted as of statistical significance. The experimental data are depicted as mean ± standard error. 

## 3. Results 

### 3.1. Synthesis of DC-Met-10

**Figure 1:**
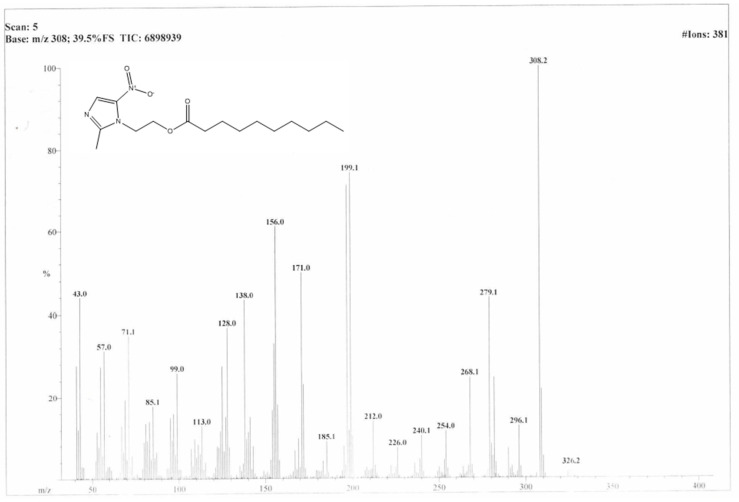
Mass spectra of “DC-Met-10”.

**Figure 2:**
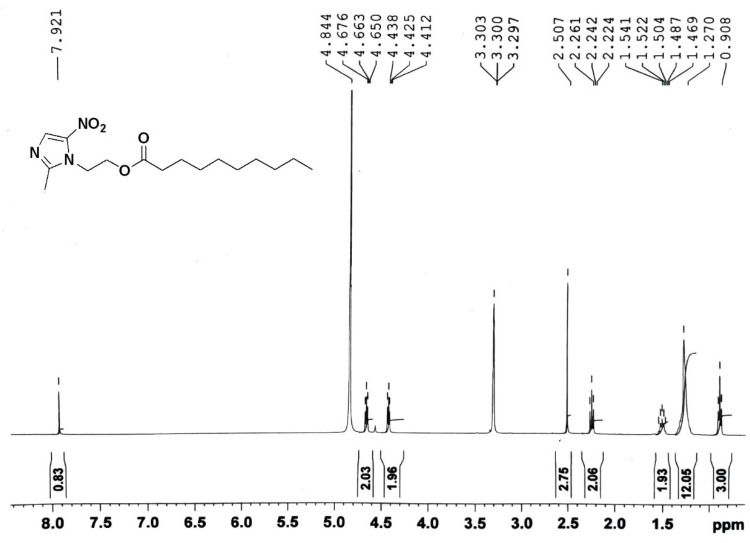
^1^H-NMR spectra of “DC-Met-10”.

Using EI-MS and ^1^H-NMR, the synthesis of DC-Met-10 was confirmed. As shown in Figure 1, the EI-MS spectra of DC-Met-10 showed a molecular ion peak at *m*/*z* 326.2, and its ^1^H-NMR spectra showed characteristic peaks of terminal methyl protons of three protons at 0.908 ppm as a triplet appeared. At 1.260–1.280 ppm, a multiplet of twelve protons of the remaining six methylene groups protons was recorded. At 2.242 ppm, a triplet of two protons of the methylene adjacent to carbonyl carbon appeared, and at 1.469–1.541 ppm, a multiplet of two protons of the remaining one methylene adjacent to the methylene of carbonyl carbon was scrutinized. At 2.50 ppm, a singlet of three protons of terminal CH_3_ attached to the heterocyclic aromatic ring was examined. At 4.425 and 4.663 ppm, a triplet of three protons of methylene adjacent to nitrogen (a part of the heterocyclic aromatic ring) and a triplet of three protons of methylene adjacent to oxygen were detected, respectively. A singlet of one proton of the heterocyclic aromatic ring was also detected at 7.921 ppm (Figure 2).

### 3.2. Particle Size, PDI, Zeta Potential, and Surface Morphology

**Figure 3:**
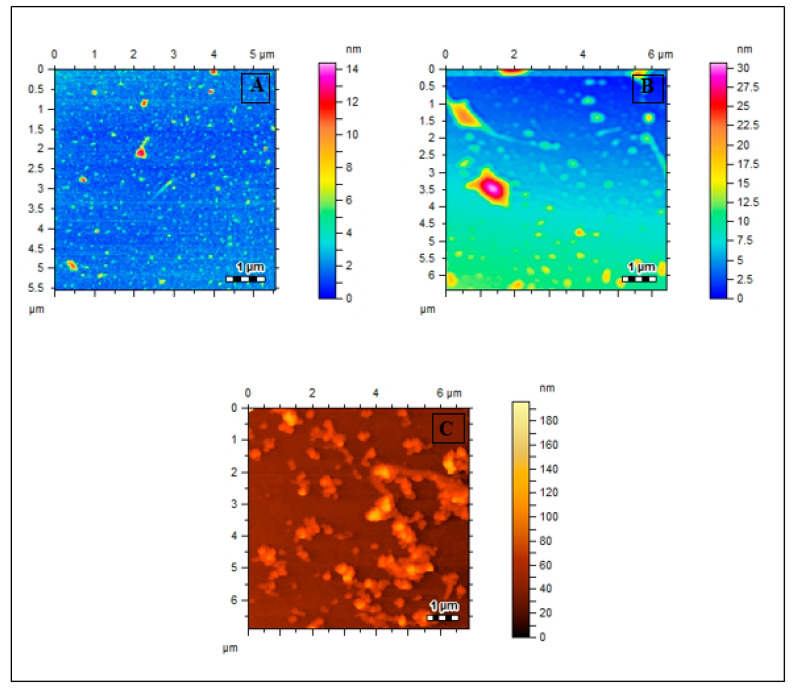
AFM images of (**A**) Met-Lip, (**B**) MOX-Met-Lip, (**C**) SUL-Met-Lip.

The results for Met-Lip, MOX-Met-Lip, and SUL-Met-Lip are shown in Table 2. Drug-loaded vesicles had greater dispersity and stability, as indicated by their PDI and zeta potential. Interestingly, drug-loaded niosomal carriers were found to be larger in size compared with empty niosomal carriers, which reflects the loading of the drug into the cavities of the vesicles [24]. The morphological evaluation showed that the drug-hosted niosomal vesicles were spherical in shape and the successive loading of the drug into the cavities of vesicles was observed. The morphology of these vesicles is depicted in Figure 3A–C, respectively. Previously, Kaskoos (2014) reported the size of moxifloxacin-loaded chitosan–dextran nanoparticles as 279.18 nm, with a good polydispersity index of 0.367 [25]. In another study, Sohrabi et al. (2016) reported the size of chitosan gel-embedded moxifloxacin niosomes to be around 290 nm [26], suggesting that this formulation has a small size and will show a good therapeutic response in vitro and in vivo.

### 3.3. % Encapsulation Efficiency of Drug-Loaded Niosomes

Drug encapsulation efficiency is an important factor as the encapsulated amount of drug in the carriers leads to constant discharge behavior and results in the enhanced bioavailability of pharmacologically active compounds [27,28]. As shown in Table 2, the developed carrier hosted a significant amount of drug and had high encapsulation. The higher encapsulation efficiency and drug-loading behavior of our synthesized non-ionic surfactant makes it a potential candidate for the delivery of active pharmaceutical agents.

### 3.4. FTIR Spectroscopy

**Figure 4:**
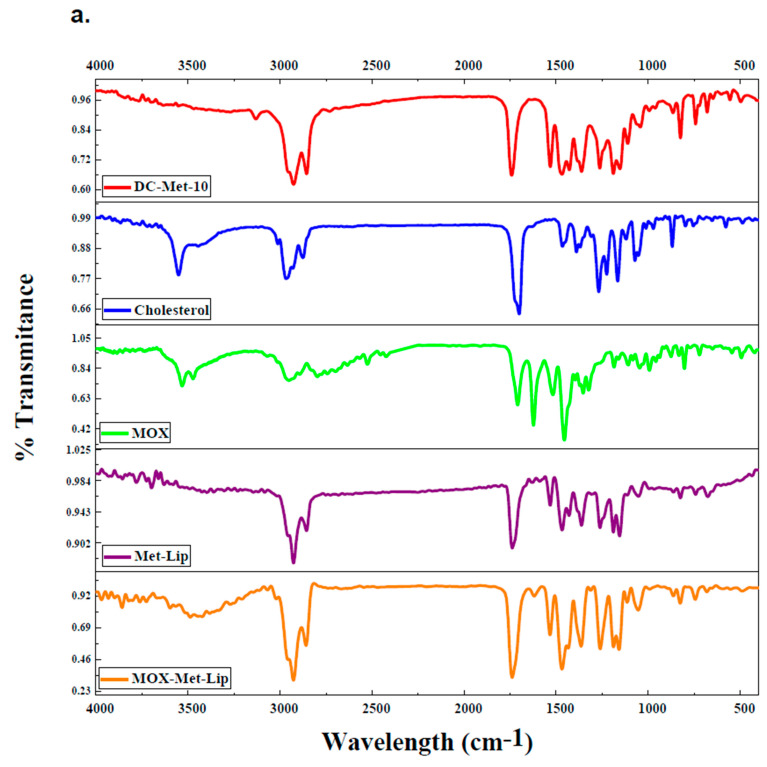

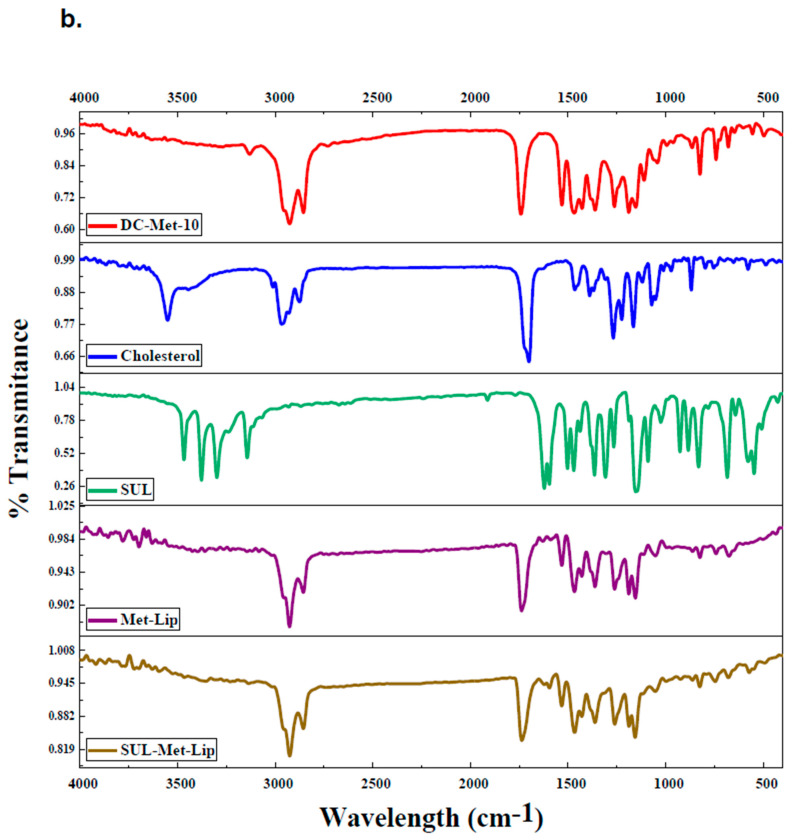
FT-IR spectra of drug-loaded nanocarriers. (**a**) FTIR of synthesized amphiphilic molecule (DC-Met-10), cholesterol, pure Moxifloxacin (MOX), without drug and drug-loaded vesicles (Met-Lip and MOX-Met-Lip), respectively. (**b**) FTIR of synthesized amphiphilic molecule (DC-Met-10), cholesterol, pure Sulfamethoxazole (SUL), without drug and drug-loaded vesicles (Met-Lip and SUL-Met-Lip), respectively.

FTIR of pure Moxifloxacin (Mox), Sulfamethoxazole (Sulp), cholesterol, synthesized amphiphilic molecule (DC-Met-10), and both drug-loaded niosomal formulations (Mox-Met-Lip and Sul-Met-Lip) and also without drug vesicles (Met-Lip) were analyzed and summarized (Figure 4a,b) [29]. In comparison to the drug alone, drug-loaded niosomes showed a slight deviation in IR frequencies, which was due to the induction of secondary interactions posed by non-ionic surfactants (Figure 4a). A broad peak pattern of the hydroxyl group (−OH) appeared at around 3200 cm^−1^ to 3500 cm^−1^ in Mox-Met-Lip, whose pattern did not appear in any other spectra. Moreover, drug peaks near 1600–1750 cm^−1^ of C=C and C=O were found to be suppressed in Mox-Met-Lip, which shows that the peaks had been enhanced in Mox-Met-Lip due to the presence of DC-Met-10 and cholesterol (Figure 4a). 

On the other hand, the peaks of Sulfamethoxazole shifted and appeared in the drug-loaded niosomal vesicles (Figure 4b). The Sulfamethoxazole peak of C=C that appeared at 1596.3 cm^−1^ was suppressed in Sul-Met-Lip at 1595.9 cm^−1^. In the fingerprint region, the drug peak of the C-O bond appeared at 1154.0 cm^−1^, and also appeared at 1157.2 cm^−1^ in Sul-Met-Lip (Figure 4b). These interactions give clear evidence about the successive loading of the drug into the cavities of niosomal vesicles and its compatibility within the vesicular system.

### 3.5. Biocompatibility Studies

Hemolysis studies are considered an important assay of nanocarriers prior to their utilization in drug delivery systems [30]. The percent hemolytic activity was found to be <10% at a maximum dose of 2 mg per mL (Table 3). This suggests that the niosomal carrier is non-hemolytic, and the niosomal carrier, even at higher concentrations, could not disrupt the biological membranes. The hemolytic activity of the niosomal carrier was observed at an accepted level of safety, as shown in Table 3.

### 3.6. Moxifloxacin- and Sulfamethoxazole-Loaded Nanocarrier Presented Potent Antibacterial Efficacy against MDR Bacteria

**Figure 5:**
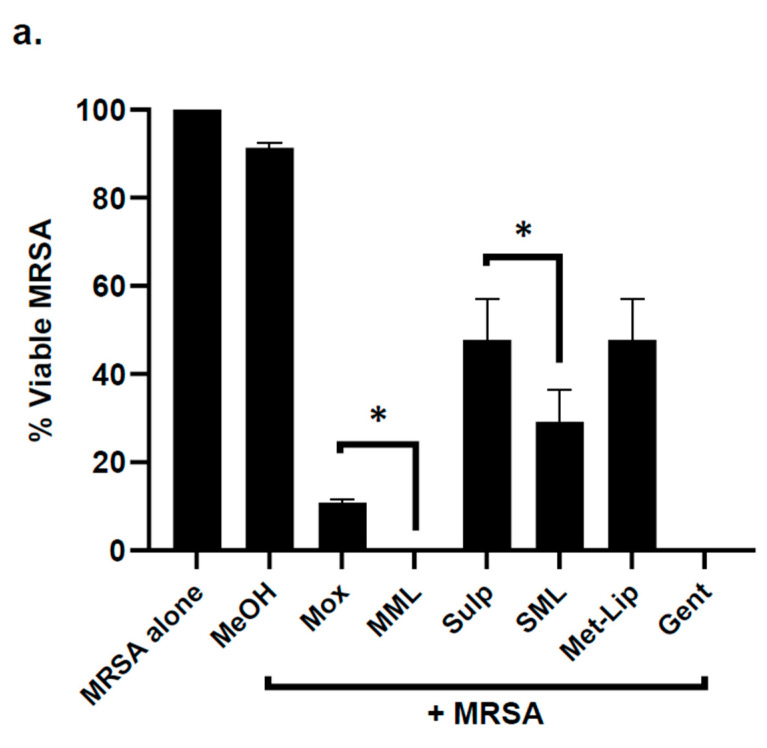

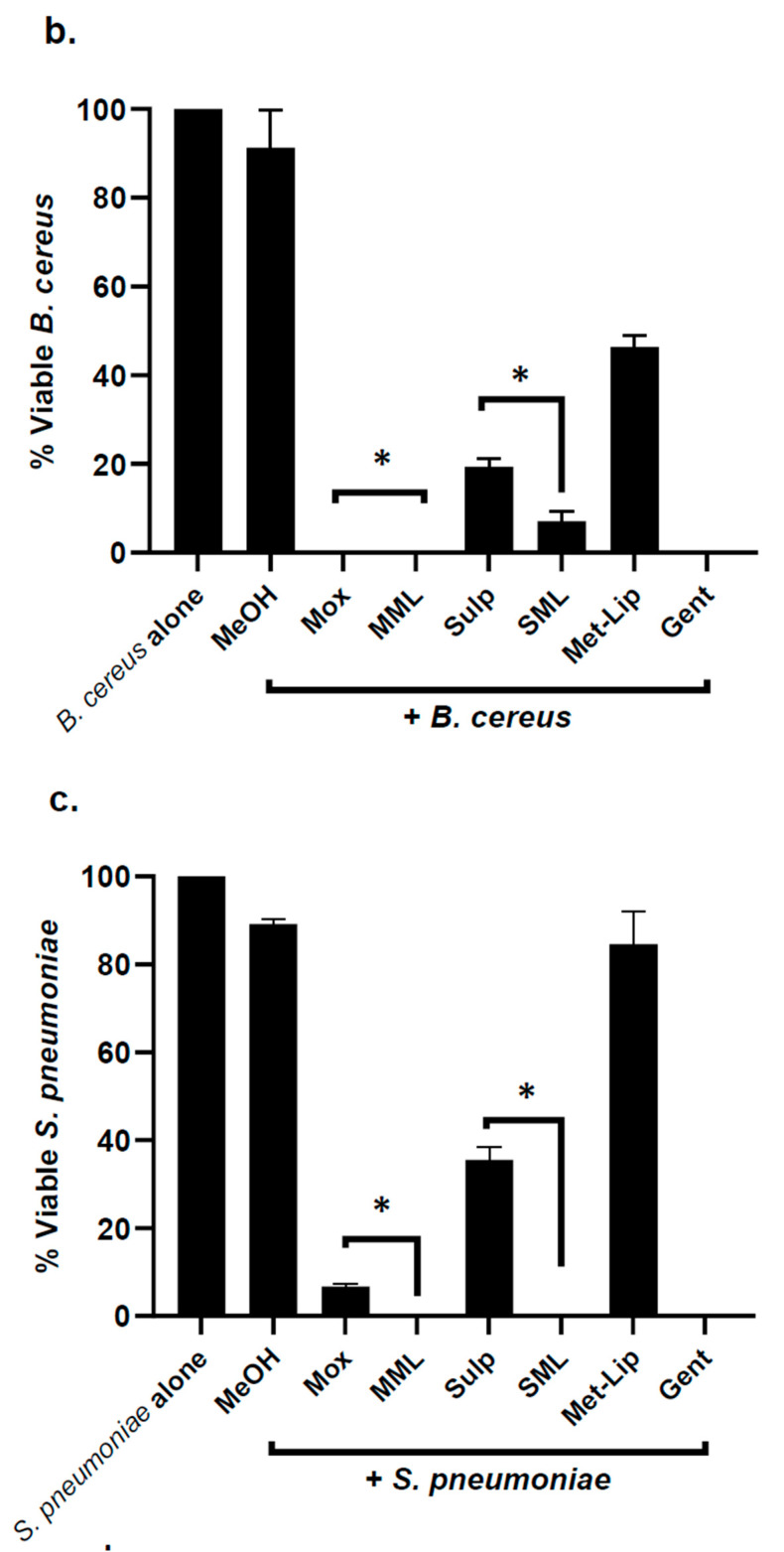

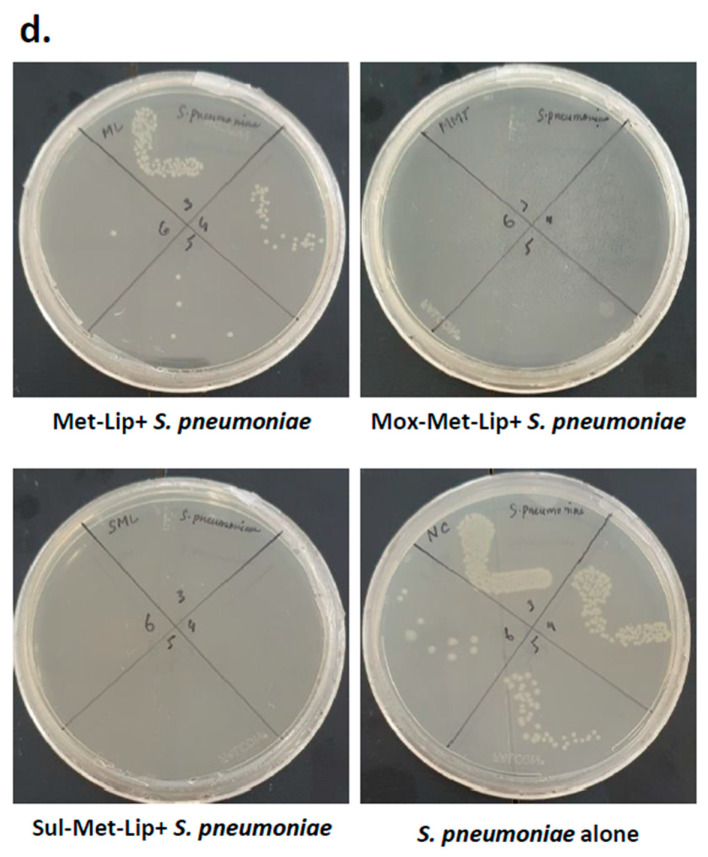
Drug-loaded nanocarriers presented significant antibacterial activity against Gram-positive clinical isolates. In brief, drugs and drug-loaded nanocarriers were incubated with one million bacteria at 37 °C for 2 h, as defined in Materials and Methods. The results showed that drugs and drug-loaded nanocarriers revealed important bactericidal activity against the Gram-positive bacteria. (**a**) When tested against MRSA, (**b**) against *B. cereus*, (**c**) *S. pneumoniae* and (**d**) illustrative effects of *S. pneumoniae.* Where MeOH represents methanol, Mox represents Moxifloxacin, MML stands for Moxifloxacin-loaded metronidazole liposomes, Sulp stands for Sulfamethaxazole, SML represents Sulfamethaxazole-loaded metronidazole liposomes, Met-Lip indicates metronidazole liposomes, and Gent represents Gentamicin. The data are expressed as the mean ± standard error of three independent experimentations performed in duplicate, where (*) represents when *p* ≤ 0.05.

Nanocarriers, drug alone, and drug-loaded nanocarriers were investigated for their antibacterial efficacy against a panel of MDR clinical isolates. The results demonstrated that the drugs and drug-loaded nanocarriers revealed significant bactericidal activity against Gram-positive MRSA (*p* ≤ 0.05, two-tailed distribution) (Figure 5a). Drug loading in nanocarriers enhanced their bactericidal activity against MRSA when compared with drug alone (Figure 5a). When tested against *B. cereus*, Moxifloxacin and Moxifloxacin-loaded nanocarriers presented exceptional antibacterial properties, with a 100% killing rate (*p* < 0.05) (Figure 5b). Similarly, Sulfamethoxazole and its counterpart nanocarriers showed significant bacterial killing activity. However, the drug-loaded nanocarriers enhanced the bactericidal effects against *B. cereus* (Figure 5b). Furthermore, the drug-loaded nanocarriers for both drugs remarkably improved the antibacterial efficacy against *S. pneumoniae* (*p* < 0.05) *(*Figure 5c).

**Figure 6:**
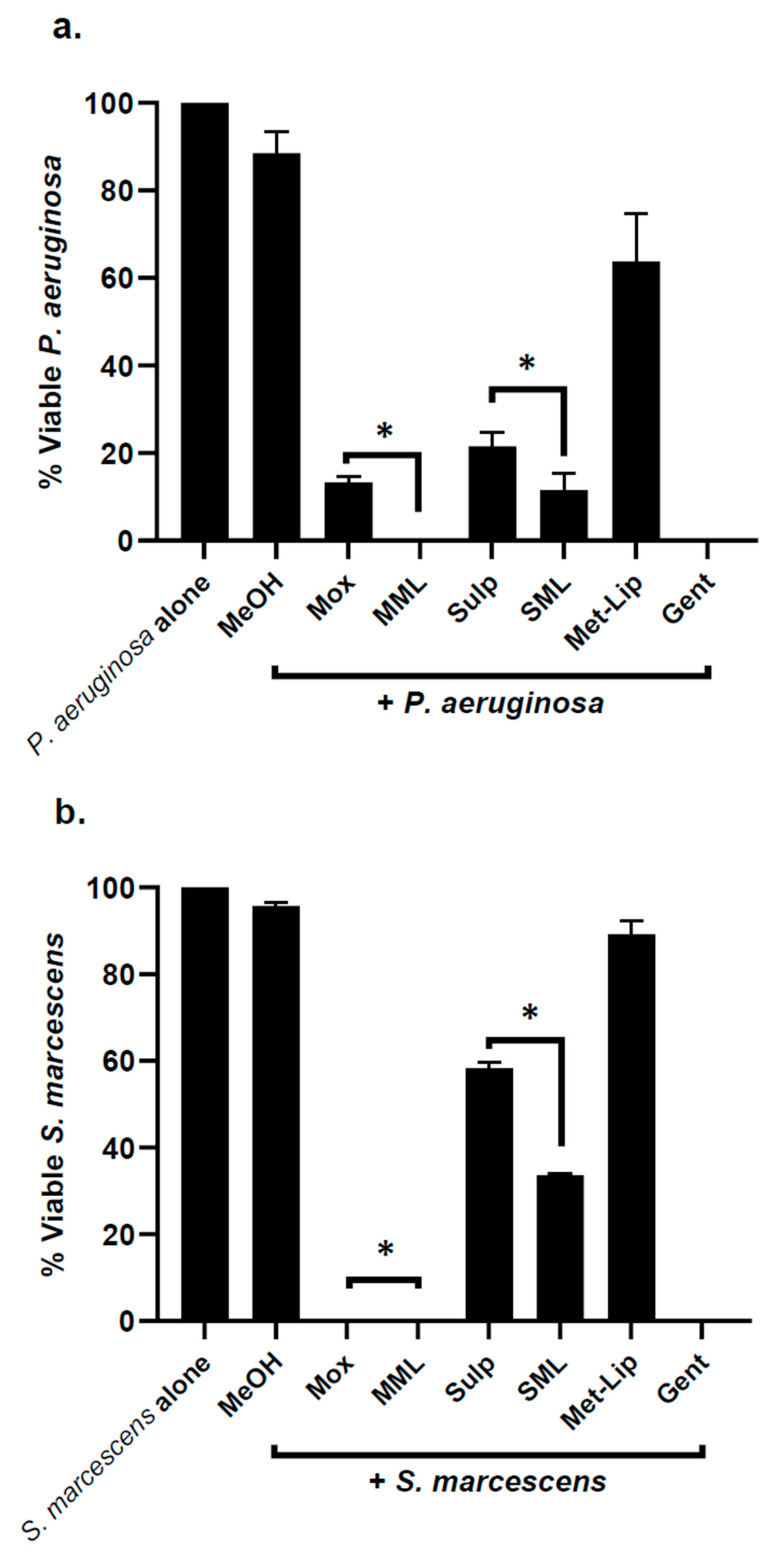

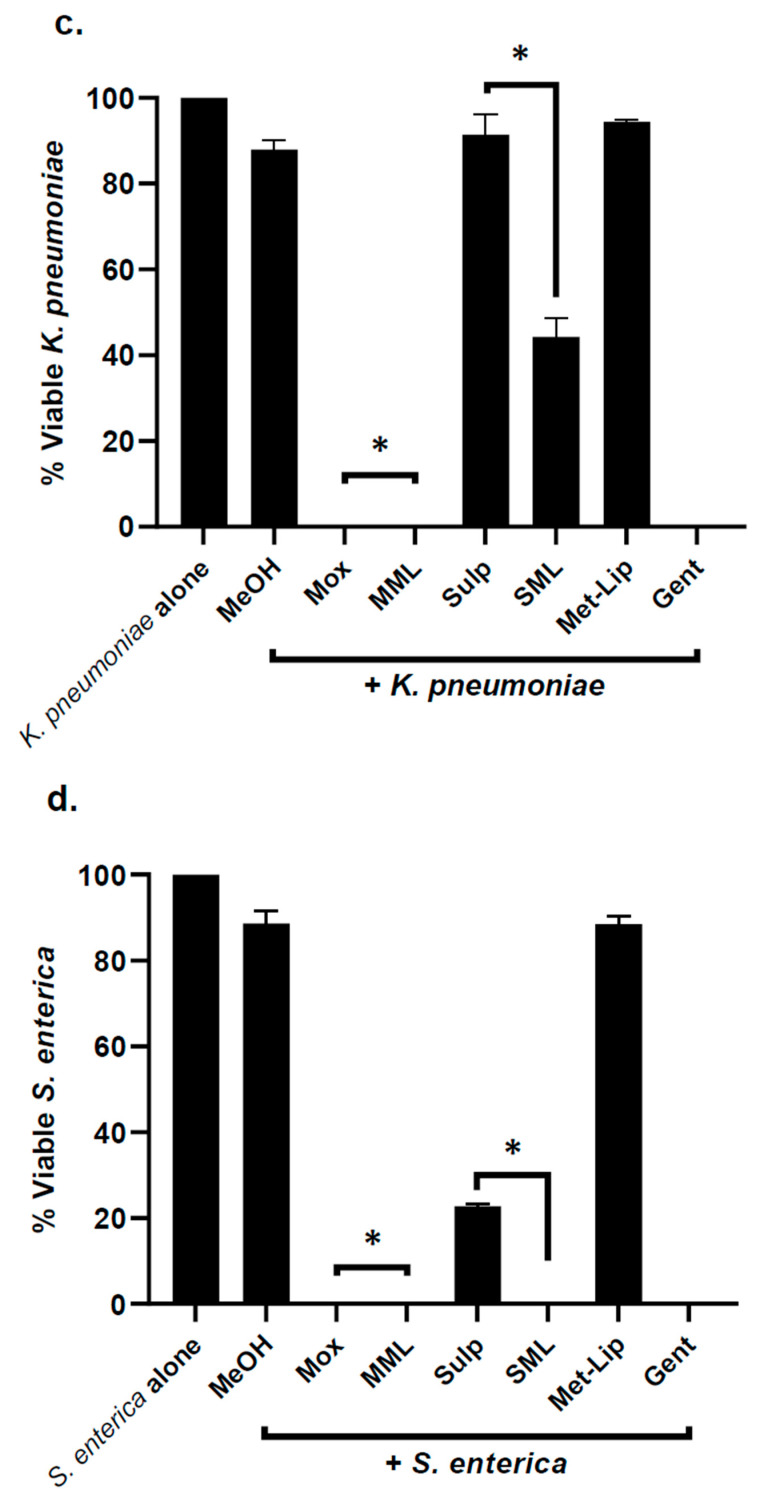

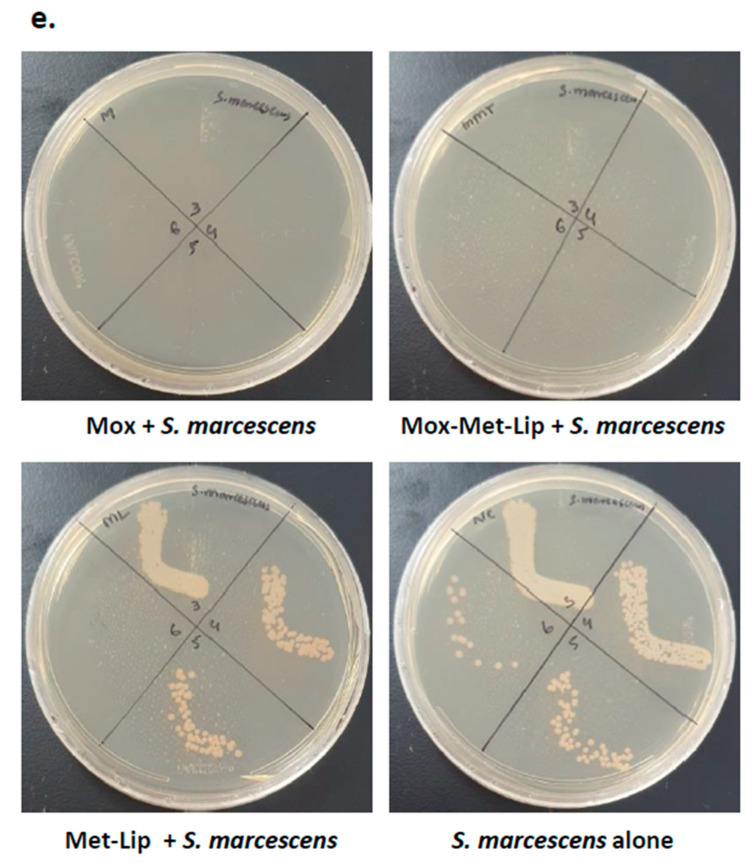
Drugs and their nanocarrier counterparts abolished Gram-negative bacteria. Briefly, bacteria (1 × 10^6^) were challenged with drugs and drug-loaded formulations for 2 h at 37 °C. Drugs and drug-loaded nanocarriers showed excellent antibacterial effects against Gram-negative bacteria. (**a**) When tested against *P. aeruginosa*, (**b**) against *S. marcescens*, (**c**) *K. pneumoniae,* (**d**) *S. enterica* and (**e**) illustrative effects of *S. marcescens.* Where MeOH represents methanol, Mox represents Moxifloxacin, MML stands for Moxifloxacin-loaded metronidazole liposomes, Sulp stands for Sulfamethaxazole, SML represents Sulfamethaxazole-loaded metronidazole liposomes, Met-Lip indicates metronidazole liposomes, and Gent represents Gentamicin. The data are expressed as the mean ± standard error, where (*) represents when *p* ≤ 0.05.

Similarly, the drug alone and drug-loaded nanocarriers were evaluated against Gram-negative clinical isolates; the results revealed that the loading of drug in nanocarriers significantly augmented bactericidal efficacy against *P. aeruginosa* (*p* < 0.05) (Figure 6a). In the case of *S. marcescens*, *K. pneumoniae*, and *S. enterica*, both Moxifloxacin and Moxifloxacin-loaded nanocarriers exhibited notable antibacterial activity, while with Sulfamethoxazole, incorporation further improved its bactericidal effects against bacteria (*p* < 0.05) (Figure 6b–d).

### 3.7. Moxifloxacin- and Sulfamethoxazole-Loaded Nanocarriers Presented Negligible Cytotoxicity

**Figure 7:**
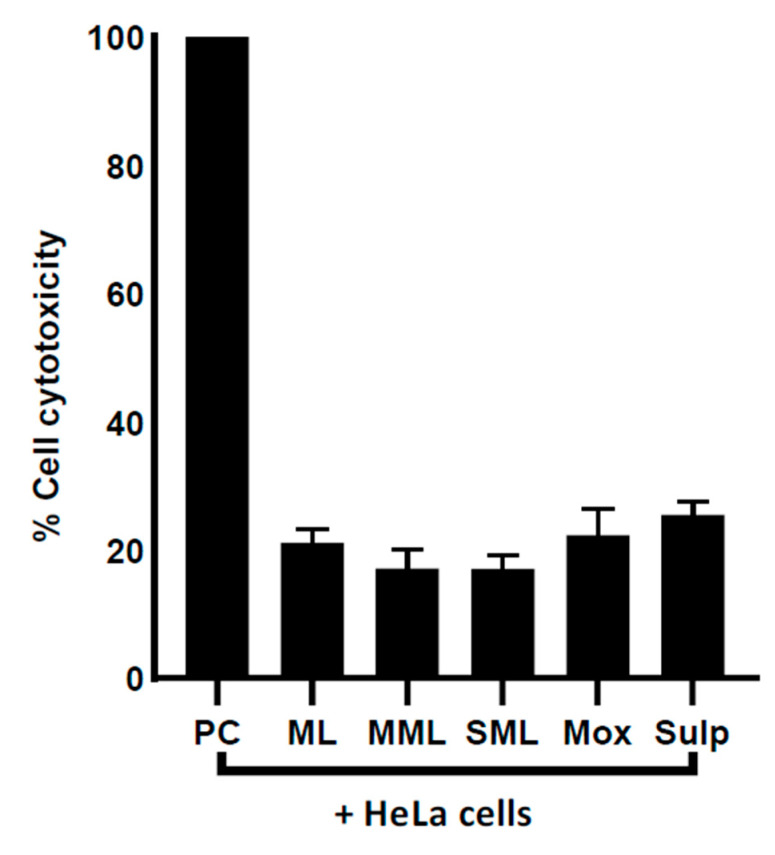
Drugs and drug-loaded nanocarriers demonstrated slight cytotoxic effects against HeLa cells. Human cells were grown in 96-well plate up to 80–90% confluency, as discussed in Materials and Methods. Next, cell monolayer was treated with drug–nanocarrier combinations overnight at 37 °C with 5% CO_2_ and 95% humidity. Where ML indicates metronidazole liposomes, MML stands for Moxifloxacin-loaded metronidazole liposomes, SML represents Sulfamethaxazole-loaded metronidazole liposomes, Mox represents Moxifloxacin, Sulp stands for Sulfamethaxazole, and PC represents positive control. The data are expressed as mean ± standard error. Data were analyzed using Graph Pad Prism software (8.0.2).

Lactate dehydrogenase assays were carried out to assess the cytotoxic effects of the drugs and drug-loaded nanocarriers. The results showed that after overnight incubation with HeLa cells, both the drugs and the drug-loaded counterpart formulations exhibited negligible cytotoxic properties (Figure 7).

## 4. Discussion

The occurrence of multi-drug-resistant bacteria is a significant challenge in the management of infectious diseases [31]. Regardless of advances in clinical practice, the global death rate caused by antibiotic-resistant bacteria has been steadily rising. The “ESKAPE” bacteria (*Enterococcus faecium*, *Staphylococcus aureus*, *Klebsiella pneumoniae*, *Acinetobacter baumannii*, *Pseudomonas aeruginosa*, and *Enterobacter* species), in addition to MDR microbes, are on the rise, making several clinically used antibiotics obsolete against the ESKAPE infections [32]. Antimicrobial resistance is anticipated to cause fatality in more than 10 million people each year by 2050 [33]. This is despite the fact that the reported literature shows significant progress in the development of antibacterial drugs [32]. Antibiotic resistance in bacteria necessitates the development of new remedies to tackle this unprecedented threat. Recent advances in engineering nanoparticles with desired physicochemical features have been hailed as a new line of defense against MDR bacteria [34]. Nanocarriers have been employed to avoid the drawbacks of traditional drug delivery systems, which include, for instance, their non-specificity, burst release, severe side effects, and human cell cytotoxicity [35]. Several types of nanomaterials, such as silver (Ag), copper oxide (CuO), chitosan, gold (Au), iron oxide (Fe_3_O_4_), titanium oxide (TiO_2_), zinc oxide (ZnO), fullerenes, NO-releasing NPs, carbon nanotubes (CNTs), and nano-emulsions, are now recognized as strong antibacterial agents [36,37,38,39,40,41,42]. In the present study, we loaded clinically approved drugs (Moxifloxacin and Sulfamethoxazole) with metronidazole-based nanocarriers and characterized them using FTIR, DLS, and AFM techniques [43,44,45]. Next, their bactericidal effects were assessed versus a panel of Gram-negative and Gram-positive MDR clinical isolates. Chemically synthesized silver/chitosan nanocomposite films loaded with Moxifloxacin exhibited broad-spectrum antibacterial efficacy against *P. aeruginosa* and *S. aureus*. This is consistent with recent studies that showed that chitosan–pullulan–silver nanocomposite films loaded with Moxifloxacin revealed antibacterial efficacies against clinical MRSA and *P. aeruginosa* [45]. An innovative CuFe_2_O4 silica-based nanocomposite loaded with Sulfamethoxazole showed extraordinary antibacterial efficacy against *S. aureus* and *E. coli* [46]. Szabó et al. (2018) demonstrated the antibacterial effects of a Moxifloxacin–β-cyclodextrin complex versus Gram-negative and Gram-positive bacteria. The complex presented significant bacteria-killing properties [17]. Lignin-based silver nanoparticles depicted notable antibacterial activity against *E. coli* and *S. aureus* [9].

Remarkably, Moxifloxacin and Sulfamethoxazole-based nanocarriers presented minimal cytotoxicity and hemolysis effects against HeLa and red blood cells, respectively. Our findings are consistent with previously published data. For instance, silver nanoparticles loaded with lignin showed negligible cytotoxic properties against human cells [9]. Similarly, cinnamic acid-based gold nanoparticles showed minimal cytotoxic properties against human cells [7]. Aseichev et al. (2014) showed that gold nanoparticles did not exhibit hemolysis effects against erythrocytes [47]. Another study revealed that magnetic Fe_3_O_4_ nanoparticles show cytotoxicity versus MCF-7 cells but do not show hemolysis activity against rabbit blood cells [30]. In conclusion, the current study showed that metronidazole-based nanocarriers loaded with Moxifloxacin and Sulfamethoxazole demonstrated notable bactericidal efficacies against MDR Gram-negative and Gram-positive pathogenic bacteria. Moreover, drug-loaded nanocarriers revealed minimal cytotoxic properties against human cells, with no hemolysis activity against human red blood cells, suggesting their probable applications as active chemotherapeutic drugs against MDR-resistant bacterial infections. Future in vivo studies, followed by clinical trials comprising drug-loaded nano-formulations, are warranted to realize these prospects and lead to the availability of much needed novel antimicrobials.

## 5. Conclusions

In summary, here, we tested Moxifloxacin and Sulfamethaxazole alone as well as loaded with metronidazole liposome nanocarriers for their antibacterial activity against several Gram-positive and Gram-negative MDR clinical isolates. Drugs alone and drug-loaded nanocarriers showed promising antibacterial effects against MDR bacteria. When compared to drugs alone, metronidazole-based nanocarriers coupled with Moxifloxacin and Sulfamethoxazole demonstrated improved bactericidal activity against numerous MDR bacteria without harming human cell lines. These findings are noteworthy and should lead to the development of new antibacterial medications with improved bioactivity. In future, such drug-loaded nanocarriers with profound antibacterial properties could be investigated in vivo utilizing a mouse/rat animal model.

## Figures and Tables

**Table 1:** Bacteria used in this study.

Bacterial Isolate	Strain
*Streptococcus pyogenes*	ATCC 49399 (clinical isolate)
Methicillin resistant *Staphylococcus aureus*	MTCC 381123 (clinical isolate)
*Streptococcus pneumoniae*	ATCC 13883 (clinical isolate)
*Bacillus cereus*	MTCC 131621 (clinical isolate)
*Escherichia coli* K1	MTCC 710859 (clinical isolate)
*Pseudomonas aeruginosa*	ATCC 10145 (clinical isolate)
*Klebsiella pneumonia*	ATCC 13883 (clinical isolate)
*Serratia marcescens*	MTCC 13880 (clinical isolate)

**Table 2:** Size, PDI, zeta potential, and encapsulation efficiency of drug-loaded niosomes.

Sample	Composition (Compound: Drug: Cholesterol)	Drug EE%	Average Vesicle Size (nm)	PDI	Zeta Potential (mV)
MOX-Met-Lip	2:2:1	73.45 ± 2.27	184.7 ± 3.61	0.375 ± 0.03	−10.1 ± 3.32
SUL-Met-Lip	2:2:1	90.35 ± 2.51	258.2 ± 5.64	0.308 ± 0.023	−14.6 ± 2.25
Met-Lip	2:0:0	0.00	149.0 ± 4.45	0.213 ± 0.032	−12.4 ± 2.5

**Table 3:** Hemocompatibility studies of synthesized amphiphilic molecule (DC-Met-10) at various concentrations ranging from 10 to 2000 µg/mL.

Concentration of Compounds (2000 µg/mL)	Hemolysis (%)
	DC-Met-10
10	-
50	-
100	-
250	-
500	-
750	-
1000	-
1250	-
1500	2.0% ± 0.06%
1750	3.5% ± 0.1%
2000	5.5% ± 0.2%

## Data Availability

The data presented in this study are available on request from the corresponding author.
